# Factors Affecting the Retention Efficiency and Physicochemical Properties of Spray Dried Lipid Nanoparticles Loaded with *Lippia sidoides* Essential Oil

**DOI:** 10.3390/biom10050693

**Published:** 2020-04-29

**Authors:** Iara Baldim, Débora M. Rosa, Claudia R. F. Souza, Raquel Da Ana, Alessandra Durazzo, Massimo Lucarini, Antonello Santini, Eliana B. Souto, Wanderley P. Oliveira

**Affiliations:** 1 School of Pharmaceutical Sciences of Ribeirão Preto, University of São Paulo, Avenida do Café s/n, Ribeirão Preto 14040-903, Brazil; iara.baldim@usp.br (I.B.); debora.rosa@usp.br (D.M.R.); souzacrf@gmail.com (C.R.F.S.); 2 CEB–Centre of Biological Engineering, University of Minho, Campus de Gualtar, 4710-057 Braga, Portugal; 3 Department of Pharmaceutical Technology, Faculty of Pharmacy, University of Coimbra, Pólo das Ciências da Saúde, Azinhaga de Santa Comba, 3000-548 Coimbra, Portugal; quele.ana@gmail.com; 4 CREA-Research Centre for Food and Nutrition, Via Ardeatina 546, 00178 Rome, Italy; alessandra.durazzo@crea.gov.it (A.D.); massimo.lucarini@crea.gov.it (M.L.); 5 Department of Pharmacy, University of Napoli Federico II, 80131 Napoli, Italy

**Keywords:** lipid nanoparticles, nanostructured lipid carriers, spray-drying, essential oil, *Lippia sidoides*, pepper rosemary

## Abstract

Essential oils (EOs) are widely used in various industrial sectors but can present several instability problems when exposed to environmental factors. Encapsulation technologies are effective solutions to improve EOs properties and stability. Currently, the encapsulation in lipid nanoparticles has received significant attention, due to the several recognized advantages over conventional systems. The study aimed to investigate the influence of the lipid matrix composition and spray-drying process on the physicochemical properties of the lipid-based nanoparticles loaded with *Lippia sidoides* EO and their retention efficiency for the oil. The obtained spray-dried products were characterized by determination of flow properties (Carr Index: from 25.0% to 47.93%, and Hausner ratio: from 1.25 to 1.38), moisture (from 3.78% to 5.20%), water activity (<0.5), and powder morphology. Zeta potential, mean particle size and polydispersity index, of the redispersed dried product, fell between −25.9 mV and −30.9 mV, 525.3 nm and 1143 nm, and 0.425 and 0.652, respectively; showing slight differences with the results obtained prior to spray-drying (from −16.4 mV to −31.6 mV; 147 nm to 1531 nm; and 0.459 to 0.729). Thymol retention in the dried products was significantly lower than the values determined for the liquid formulations and was affected by the drying of nanoparticles.

## 1. Introduction

Essential oils (EOs) are composed by volatile, natural, and complex hydrophobic compounds produced by the secondary metabolism of plants [1,2,3,4,5]. They represent a green alternative for diverse industrial sectors, such as pharmaceutical, food, cosmetics, health, agriculture, and livestock, given their proven biological activities as bactericidal, antiviral, fungicide, insecticidal, antioxidant, and other activities [6,7,8,9,10,11,12,13,14]. Nevertheless, EOs might present problems of instability when exposed to heat, moisture, oxygen, and light. Besides, the reduced water solubility of EOs is another limitation for their incorporation in more elaborated products [15,16]. Encapsulation technologies represent an effective approach to preserve the physicochemical and biological properties of EOs. Their loading into nanoparticles enhances their stability against environmental factors, reduces their volatility, modulates the release, with the possibility to reduce toxicity and increase bioavailability [17,18].

Encapsulation into lipid nanoparticles is receiving significant attention in the pharmaceutical and food sectors, as they offer several advantages, namely their biocompatibility, biodegradability and low toxicity, the possibility to encapsulate hydrophilic and lipophilic compounds, low production costs and easy scale-up [19,20,21,22,23]. Lipid nanoparticles are classified into different types produced by very different lipids and by different methods [24,25]. Among them, nanostructured lipid carriers (NLCs) receive special attention as, due to their solid matrix, they exhibit modified release profile and site-specific targeted delivery [26,27]. Besides, the presence of liquid lipid (oil) in their composition gives the additional advantage of enhanced drug-loading capacity [23]. As NLCs also have the capacity to load a range of nutraceuticals for oral administration [28], these particles hold a remarkable potential as a delivery system to build up the emerging area of the nanonutraceuticals with improved safety and efficacy [24,29,30].

In most situations, the production of a dried formulation from a liquid dispersion may be an advantage or even recommended to improve the stability of the payload [31,32,33]. A powdered product—obtained either from freeze-drying or spray-drying—exhibits higher shelf-life. To obtain a free-flowing powdered product, spray-drying is nevertheless more cost-effective then freeze-drying or lyophilization, as it is a faster drying process and is less expensive. Advantages such as the single-stage method and the continuous progress of the process make this technique widely used in the industry [34]. Some studies report that the type of biomaterial, core–wall ratio, drying parameters, and storage conditions affect the microstructure of spray-dried microcapsules and the retention of the loaded drug. On the other hand, product microstructure affects the functionality, stability, and flow properties of the particles [35,36].

*Lippia sidoides*, belonging to the genus *Lippia* [37,38], is a typical herb species of Northeast Brazil widespread used in traditional medicine; it is known in Brazil as “alecrim-pimenta” (pepper-rosemary). The main bioactive compounds in the EO of *Lippia sidoides* are monoterpenes, like thymol, *p*-cymene, myrcene, and the sesquiterpene, as caryophyllene [39,40,41]. These compounds are responsible for the biological properties of this EO, i.e., antimicrobial, antinociceptive, anti-inflammatory, and antioxidant activities [42,43,44].

This study aimed at investigating the effects of the lipid matrix composition and drying process (spray-drying) on the thymol retention efficiency and on the physicochemical properties of the NLCs loaded with *Lippia sidoides* (pepper-rosemary) EO.

## 2. Materials and Methods

### 2.1. Material

*Lippia sidoides* EO was purchased from PRONAT (Produtos Naturais do Nordeste LTDA, Horizonte, Ceará, Brazil). The constituents of the NLCs were Gelucire 50/13 (Gattefossé, Saint-Priest, France), oleic acid (LabSynth, Diadema, São Paulo, Brazil), Labrasol (Gattefossé, Saint-Priest, France), Span^®^ 80 (Sigma-Aldrich, Darmstadt, Germany), Tween^®^ 80 (LabSynth, Diadema, São Paulo, Brazil), and Poloxamer 188 (Kolliphor P 188 Micro, kindly donated by BASF, Ludwigshafen, Germany). Mixtures of whey protein (Arla Foods Ingredients S.A., Buenos Aires, Argentina), arabic gum (Nexira, São Paulo, Brazil), and colloidal silicon dioxide (Aerosil 200, Evonik Degussa, Essen, Germany) were used as spray-drying carriers. ultrapure water (Direct Q System, Millipore, Merck KGaA, Darmstadt, Germany) was used in the preparation of NLCs. Other chemicals and solvents used were of pharmaceutical or of high performance liquid chromatography (HPLC) grade.

### 2.2. Characterization of the Major Constituents of the Lippia sidoides Essential Oil by Gas Chromatography Coupled to Mass Spectrometry

The main constituents of *Lippia sidoides* EO were characterized by gas chromatography coupled to mass spectrometry (GC-MS Shimadzu QP-2010, Kyoto, Japan), using an DB-5 (30 m × 0.25 mm × 0.25 µm) capillary column. The chromatographic conditions were based on previous study [19]. Hydrogen was used as the carrier gas, temperature-programmed from 60 °C to 240 °C with a ramp of 3 °C min^−1^. Other chromatographic conditions were as follows: injector temperature of 240 °C, H_2_ with a flow rate of 1.30 mL/min as carrier gas, detector temperature of 260 °C, and split ratio 1:50. The constituents of the EO were identified by comparing the mass spectra obtained with those spectra published in the equipment database (Wiley electronic library or WILEY7.LIB) and by comparison with the Kovats index (IK), relative to a series of *n*-alkanes (C9-C20 [45]) with the ones reported by Adams [46].

### 2.3. Preparation of Nanostuctured Lipid Carriers

Eight distinct NLC formulations were prepared according to Table 1, by the phase inversion method. Gelucire 50/13 plus the liquid lipids oleic acid or Labrasol comprised the lipid phase, and poloxamer 188 or a combination of Tween^®^ 80:Span^®^ 80 at a ratio 1:1 were added to the aqueous phase. Whey protein (P) and arabic gum (G) mixtures were used as drying carriers. Small amount of colloidal silicon dioxide (SiO_2_) was added only to emulsions having a high G:P ratio intending to improve drying performance. Gelucire and the liquid lipids were mixed and heated 10 °C above the Gelucire melting point (≈50 °C). The surfactants were dissolved in ultrapure water and heated to the same temperature of the lipid phase. The heated EO was slowly added to the lipid phase, under magnetic stirring. Finally, the aqueous phase was homogeneously dispersed into the lipid phase with EO by using a high-speed stirrer (Ultra Turrax T18, IKA, Wilmington, NC, USA) at 18,000 rpm/min for 2 min. The oil-in-water (O/W) emulsion formed was sonicated by an ultrasonic sonicator VCX-750 (SONICS Vibracell, Newtown, CT, USA), with aid of 13 mm probe at a frequency of 20 kHz at an intensity of 70% for 2 min. Then, the drying carrier, previously hydrated was added. Solids concentration of formulations was 16.7% *w/w* for samples coded as A1 code and 21.4% *w/w* for A2 code samples.

### 2.4. Spray-Drying and Drying Performance

The resulting lipid-based systems loaded with *Lippia sidoides* EO were dried in a Lab-Plant SD-05 spray dryer (Lab-Plant Ltd., Huddersfield, UK). Operating conditions were as follows: feed rate 4 g/min, inlet temperature 100 °C for the formulations with A1 code and 90 °C for the formulations A2 code, diameter of the atomizer nozzle 1 mm, drying gas flow rate 60 m^3^/h, and atomizing pressure and gas flow of 3 bar and 17 L min^−1^, respectively. The spray-drying performance was evaluated by determining the product recovery, calculated as the amount of powder collected from the dryer divided by the total amount of solids fed. Samples of the spray-dried powders were saved for the characterization of the physicochemical properties and quantification of thymol retention in HPLC.

### 2.5. Zeta Potential, Emulsion Size, and Polydispersity Index of the Liquid and Redispersed Dried Systems

Zeta potential, particle size, and polydispersity index of the liquid and redispersed dried lipid systems were determined by dynamic light scattering (DLS) using a Zetasizer Nano–ZS90 (Malvern, UK). The dried samples were redispersed in ultrapure water at the same concentration of the liquid sample, stirred for 30 min, and diluted at a ratio of 1:200 (*v/v*) before the measurements. The measurements were made in triplicate.

### 2.6. Flow Properties

The flow properties of dried powders were assessed through the determination of Hausner Ratio (I_Hausner_) and Carr’s Index (I_Carr_), according to Equations (1) and (2).

(1) $$I_{Hausner=\frac{\rho_{t,\;1250}}{\rho_{b}}}$$

(2) $$I_{Carr}=\frac{\rho_{t,1250}-\rho_{b}}{\rho_{t,\;1250}}\times 100$$

where *ρ_b_* is the freely bulk density (*ρ_b_* = m_0_ V_0_^−1^), *ρ*_*t*,1250_ is the tapped bulk density, determined using the volume occupied by the powder after tapping the probe 1250 times from a distance of 14.0 mm (USP, 2007) [47]. A Caleva Tapped Density Tester Type TDT (Frankfurt, Germany) was employed to measure bulk and tapped densities.

### 2.7. Moisture Content and Water Activity

The moisture content was determined immediately after drying by Karl Fischer titration, using 100 mg of powder in a Karl Fischer 870 Titrino Plus Methrom (Herisau, Switzerland), calibrated with water before analysis. The water activity of the powders was determined in triplicate using an Aqua Lab 4Tev^®^ water activity meter (Decagon Devices, Pullman, WA, USA) using the capacitance electrode. The measurements were made in triplicate, and the results expressed as mean and deviation.

### 2.8. Thymol Retention of Nanostructured Lipid Carriers and Spray-Dried Product

The GC-MS characterization of the *Lippia sidoides* EO confirmed thymol as the most abundant compound. Therefore, it was used as a marker to monitor the process development. High-performance liquid chromatography with diode array detection (HPLC-DAD) was employed to monitor the concentration of thymol in the liquid and spray dried lipid systems. Chromatographic conditions were based on the method proposed by Leal et al. [48], with some modifications [49]. Analyses were performed in an HPLC Shimadzu Prominence LC-20A series and an LC-6A double pump (Shimadzu Corporation, Kyoto, Japan) using a C-18 column (Shimadzu Shim-Pack CLC(M) 4.6 mm × 25 cm, 5 µm, 100 Å) at 30 °C. The mobile phase was a gradient of water (A) and acetonitrile (B). The acetonitrile concentration was changed as follows: 0–2 min, 10% B; 2–7 min, linear increase of B to 78%; 7–17 min, 78% B; 17– 20 min, linear increase of B to 100%; 20–23 min, 100% of B; 23–26 min, linear decrease of B to 10%, 23–32 min, 10% of B. The chromatograms were recorded at 276 nm. The method of sample preparation consisted of diluting the samples in methanol, homogenize the mixture in the ultrasound bath and keep the solution under magnetic stirring for 30 min. After the extraction, the samples were centrifuged for 5 min at 5000 *g*. The supernatant was filtered through a membrane of 0.45 µm pore size, and 20 µL were injected in the chromatograph for quantification.

### 2.9. Particle Morphology

Photomicrographs of the spray dried-powder morphology were acquired by scanning electron microscopy with field emission gun (SEM-FEG). A small quantity of powder was mounted on stubs with double-sided adhesive carbon tape. Each sample was coated with platinum and analyzed in an Inspect F-50 (FEI, Eindhoven, The Netherlands) at 5 kV.

### 2.10. Statistical Analysis

Statistical analysis was performed for some assays by using one-way ANOVA followed by Tukey’s post hoc test to determine if there are significant differences between the means.

## 3. Results and Discussion

The GC-MS results permitted the detection of twenty-seven compounds of the *Lippia sidoides* EO, and 26 were successfully identified (see Table 2), corresponding to 99.94% of the EO composition, as reported previously by us [19] and according to the equipment library. Thymol, the bioactive compound linked to most of the biological activities of *Lippia sidoides* [50], was confirmed as the most abundant component, with a concentration of 68.2%, as previously reported in the literature [51,52,53]. The benzene, 1-methyl-4-(1-methylethyl)- (9.43%), the trans-caryophyllene (7.72%), β-myrcene (2.84%), and the γ-terpinene (2.71%) are other main constituents of EO. Altogether, these five compounds reach 90.92% of the *Lippia sidoides* EO composition.

Product recovery was the parameter used to evaluate the drying performance. The average value of product recovery measured for spray-dried products having the lower amount of drying carrier (small G:P ratio, A1 code) was 44.7 ± 2.0%; while average product recovery reached the value of 50.9 ± 3.4%, for the formulations with a high amount of drying carriers (high G:P ratio (A2 code). The stickiness of the atomized material to the spray-drying chamber was observed during all drying runs. Formulations with A1 code (spray-dried at 100 °C) exhibited high adhesion of the product to the drying chamber. For this reason, the spray-drying temperature was decreased to 90 °C to dry the formulations with A2 code, and a small amount of colloidal SiO_2_ was added (1%) to the drying carriers. Although a slight increase in product recovery was observed, the stickiness problems remained. According to Cortés-Rojas et al. [54], the stickiness of the powders to the drying equipment is one of the major causes of the product losses, especially when working with materials having low glass transition temperatures, as is the case of the lipid-based formulations. The analysis of the results did not evidence relevant differences in product recovery by changing lipids and surfactant systems in the formulations.

Zeta potential (Z), emulsion size (dp), and polydispersity index (PI) are important parameters generally used to characterize dispersed systems, including emulsions, solid lipid nanoparticles and nanostructured lipid carriers [21,22,55,56].

Zeta potential (Z) characterizes the particle surface charges and thus provides information on the repulsive forces between particles and droplets. It is a very useful parameter for the assessment of the physical stability of colloidal dispersions. Generally, Z value more than +20 mV or less than −20 mV promotes the repulsion of the particles from each other, predicting good physical stability of nanoparticle dispersion [20,57].

**Figure 1:**
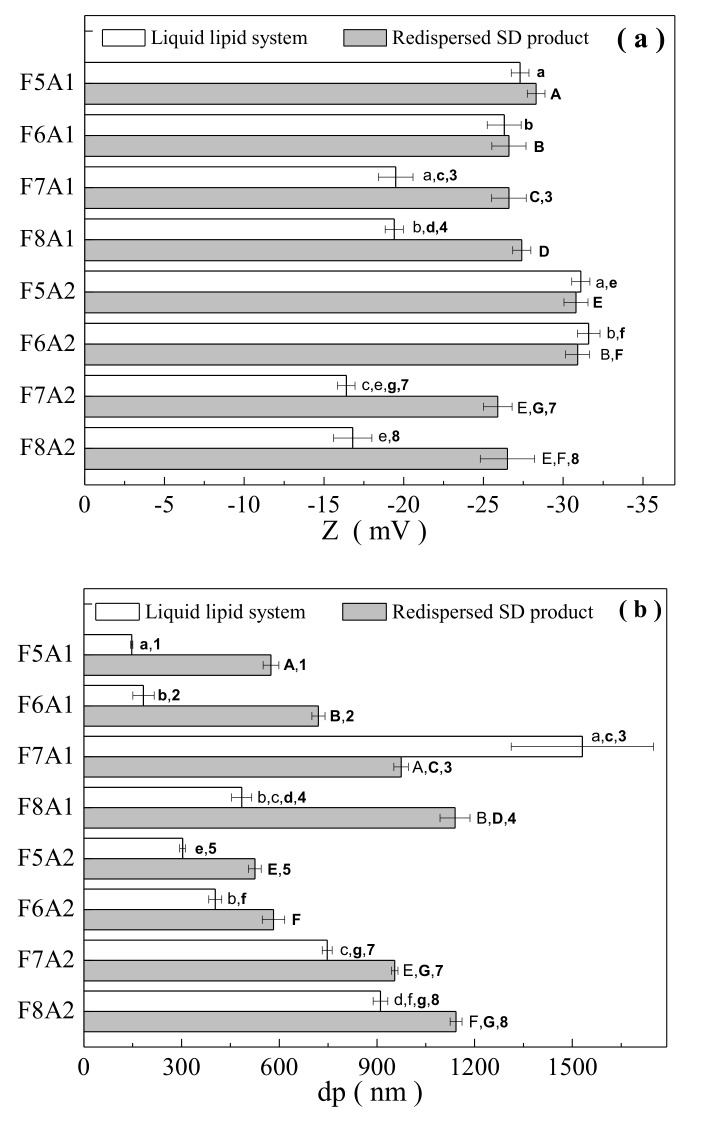

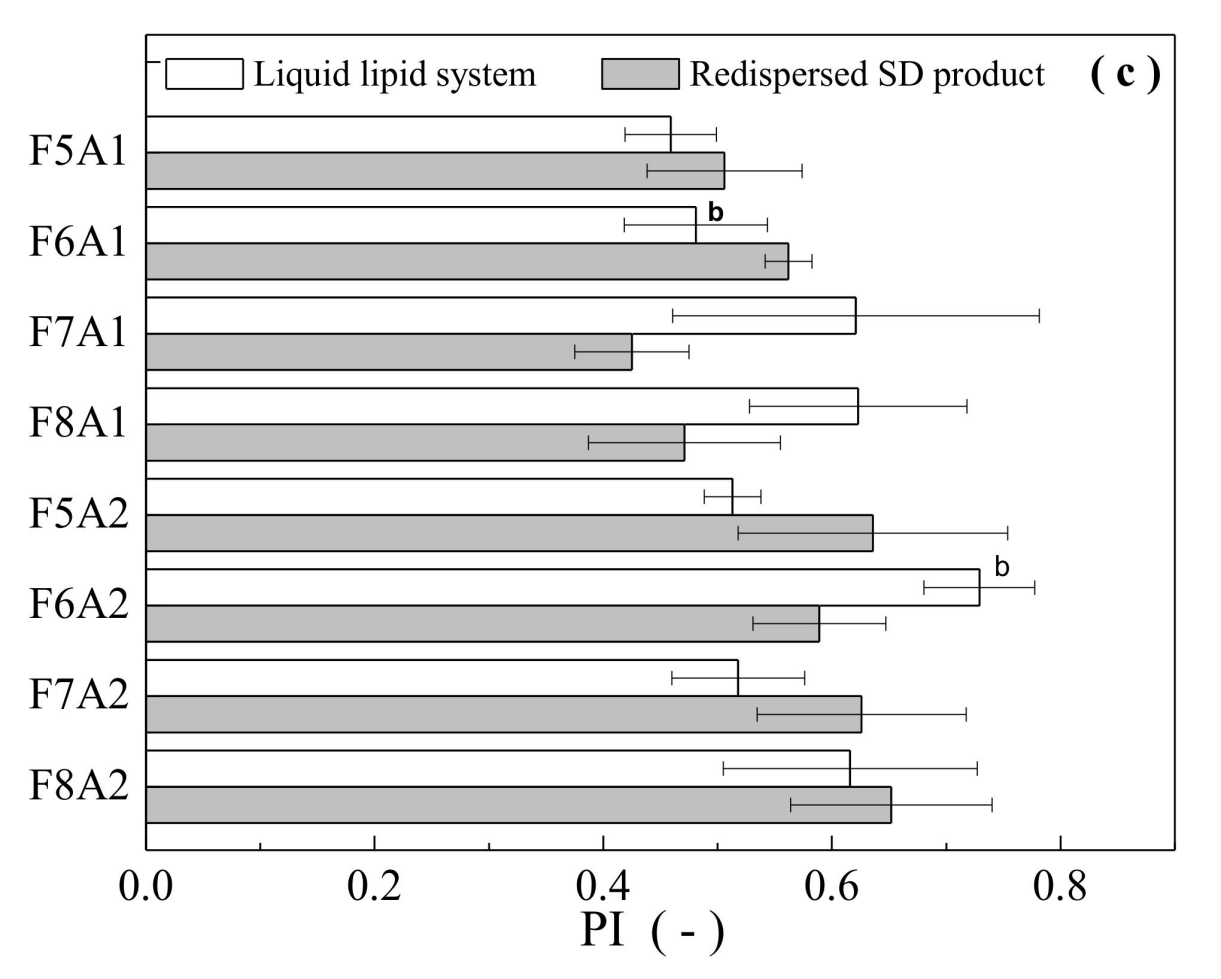
Comparison between the experimental results of zeta potential (Z), emulsion size (dp), and polydispersity index (PI) of the liquid and redispersed dried lipid systems, (**a**–**c**), respectively. Same letters/numbers indicate a statistically significant difference (Tukey’s post-test, *p* ≤ 0.05).

Figure 1a–c shows respectively the comparison between the experimental values Zeta potential (Z), particle size (dp) and polydispersity index (PI) of the lipid-based systems, before and after spray-drying (redispersed at same initial concentration). Z, dp and PI of the redispersed dried product falls between −25.9 mV and −30.9 mV, 525.3 nm to 1143 nm, and from 0.425 to 0.652, respectively; showing slight differences with the ones measured for liquid NLCs (from −16.4 mV to −31.6 mV; from 147 nm to 1531 nm; and from 0.459 to 0.729). Analyzing each formulation, in general, the zeta potential, particle size, and polydispersity index values are slightly higher in the dried form when compared to liquid composition.

One-way variance analysis followed by Tukey’s post hoc test was carried out for the experimental results in order to verify statistically significant differences due to encapsulating formulation constituents and drying.

The ANOVA of the data from Figure 1a indicates that the changing the surfactant system from Tween^®^ 80:Span^®^ 80 (hydrophilic-lipophilic balance of 9.7) to Poloxamer 188 (hydrophilic-lipophilic balance ≈ 29.0), although their distinct physicochemical properties, did not evidence significant effect on the zeta potential both for the liquid and spray dried redispersed samples. This behavior can be attributed to the small percentage of these compounds regarding the other lipids and drying carriers used and by the similar HLB presented after their respective mixtures with Gelucire. On the other hand, the type of liquid lipid affects significantly the zeta potential of the NLCs. Formulations containing Labrasol showed smaller values of zeta potential, independent of the drying carrier systems used. The differences encountered in the zeta potential values between oleic acid versus Labrasol based NLC may also be attributed to the different capacity of these latter in solubilizing the essential oil. Considering that essential oil has been dissolved within 30 min stirring, Labrasol may have a lower solubilizing capacity than oleic acid.

For the redispersed spray-dried powders, the reduction on zeta potential was only verified for the sample containing the drying carrier with a higher G:P ratio (*p* ≤ 0.05). Nevertheless, for the redispersed powders, the comparisons are trickier, since other factors such as the degree of product redispersion and the EO retention as well, affects the zeta potential results. Indeed, compared with the blank system, the addition of EO causes a significant reduction in zeta potential. Therefore, the distinct values of EO retention in the spray-dried powders, undoubtedly affect the experimental values of zeta potential.

Concerning the drying carriers’ systems, there are significant differences between the zeta potential determined for both systems (coded as A1 and A2). For NLCs containing oleic acid, a slight increase in zeta potential might be verified with the increasing the G:P ratio (and consequently solids content—A2 code samples) of the drying carrier systems. A different behavior could be seen for NLCs containing Labrasol. For redispersed spray-dried products, most of the samples did not evidence a statistically significant effect of the drying carrier system. Again, the results for dried powders are more difficult to analyze, since differences of the EO loaded in the product and the redispersing operation would affect in any way the measured results.

By comparing the experimental values of zeta potential of the original and redispersed spray-dried products, no differences are verified for the formulations containing oleic acid, independent of other factors. However, significant increase due to the spray-drying process can be observed by those containing Labrasol.

In summary, considering the zeta potential analyses, it can be supposed that the system containing oleic acid is more stable compared to Labrasol. From the technological point of view, all the systems were considered stable, not showing any kind of instability during accelerating stability testing of the NLCs.

Figure 1b shows that the spray-dried powders exhibited particle sizes in the nanoscale range, with the exception of both F8 formulations. Larger particle sizes were exhibited for Labrasol containing formulations comparatively to the ones composed by oleic acid, independent of the drying carrier system used (*p* ≤ 0.05). This behavior was observed for both liquid and spray dried products. The changing in the surfactant systems added to the formulations (Tween^®^ 80:Span^®^ 80 or Poloxamer 188), presented significant effect only for the Labrasol-containing formulations, for the drying carrier system with low G:P ratio, as can be seen in Figure 1b.

In general, the increase of the G:P ratio (and solid contents as well) leads to a bigger particle size. These effects were evident for NLCs, although they were not observed for the redispersed spray-dried products. We speculate that NLCs should present a smaller droplet diameter compared to the redispersed spray-dried powders. This behavior is observed for almost of the data shown in Figure 1b, except for F7A1 and 7A2, which exhibited an unpredicted behavior.

The polydispersity index (PI) indicates the width of the particle size distribution, which ranges from 0 to 1. Theoretically, monodisperse populations exhibit PI = 0 [58,59,60]. According to Maitani et at. [61], a polydispersity index value greater than 0.3 shows a high degree of heterogeneity. In this work, the PI values obtained were around 0.5, indicating particles with a slight heterogeneous distribution. In this work, the formulation variables and spray-drying did not show statistically significant effects on PI. The unique exception was observed for the formulations F6A1 and FA62, where an increase in PI was observed by increasing the G:P ratio (and solids content) of the drying carrier.

The experimental results of the moisture content, water activity, bulk and tapped powders densities (ρ_b_ and ρ_t,1250_), I_Hausner_, and I_Carr_ of the spray-dried powders are presented in Table 3.

Moisture content and water activity are parameters related to the composition of the formulations and drying conditions employed [54,62,63]. Moisture content is related to the total quantity of water in the dried system [64]. Water activity and moisture content are optimally present for the stability and safety limit of the product. Although the statistical analyses of the experimental results of product moisture content showed some significant effects due to changing formulation parameters namely surfactant systems, liquid lipids, and drying carriers’ mixture, the differences observed are not technological relevant. The measured results ranged from 3.78% to 5.20%, which are adequate to ensure product quality. The water activity of dried powders is a more pertinent factor than the moisture content. It is linked to the amount of free water content available for microbial growth and other product degradation reactions. Although factors such as storage temperature and pH can affect the growth of spoilage microorganisms and food-borne pathogens, the water activity is generally recognized as being most important [65]. The water activity may vary between 0 and 1, and higher values imply a great amount of water available for physicochemical degradation reactions and microbial growth. Water activity values below 0.5, prevent microbial growth [66]. The water activity was not measured for the powders with A1 code, but all the values obtained for the A2 powders were below the safety limit of 0.5.

The flow parameters, I_Hausner_ and I_Carr_, were calculated from the experimental values of tapped and bulk densities of spray-dried powders. I_Hausner_ and I_Carr_ are factors commonly used as an indirect measure of particle flowability. Both are based on the friction and accommodation of the particles, which in turn are influenced by the physicochemical parameters of the particles, such as size and surface properties. Low I_Hausner_ and I_Carr_ values indicate low cohesiveness, therefore better flow [54]. Powders presenting low friction between the particles, such as large particles, have an I_Hausner_ around 1.2, whereas values higher than 1.6 are presented by smaller and cohesive particles with restricting flow. Materials with I_Carr_ between 5% and 15% are assumed of excellent fluidity; from 12% to 16% good flow; from 18% to 21% scarce; from 22% to 35% weak flow; from 35 to 38% very weak; and higher than 40% extremely weak flow. Results presented in Table 3 show that the dried products presented weak and very weak flow, usual for spray-dried powders, normally linked to the small powder particle diameters obtained. The samples composed by Labrasol and Poloxamer 188 (F8A1 and F8A2) exhibited better flow properties. It is concluded that the flow properties have an inversely proportional relationship with the diameter of the particles since these were also the samples for which the particles showed the largest size.

**Figure 2:**
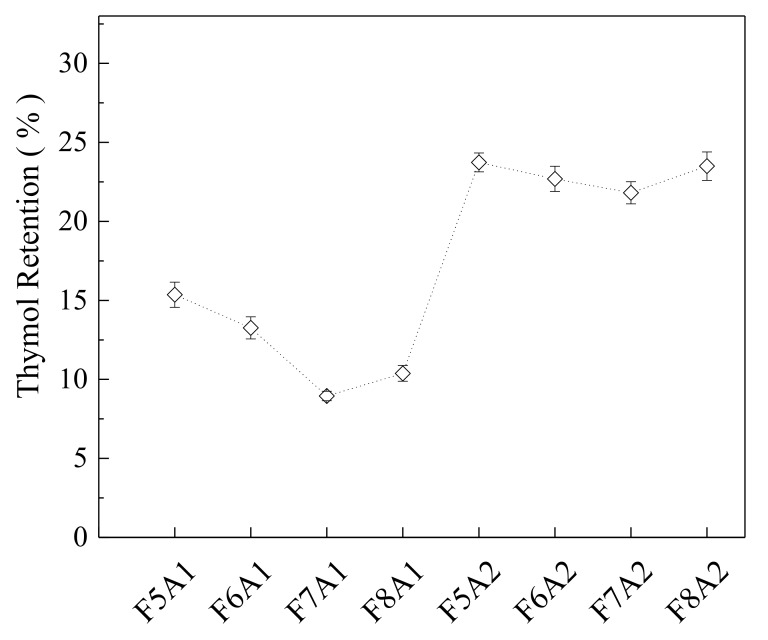
Retention of thymol in the spray dried product. Percentage relative to the initial concentration of thymol in essential oil.

The volatiles retention is governed by factors linked to the physicochemical properties of the volatile compound, the constituents of NLC, the drying carriers (wall material), and drying conditions [53,67,68]. It is well known in the literature that high total solids content in the encapsulating emulsions are beneficial to volatile retention [35,53]. It can be explained because high volatilities, at low concentrations, tend to evaporate much faster from aqueous solutions than water does. However, if the solution or suspension concentration is sufficiently high, the reverse is generally true [69]. This trend was also observed in the results obtained for thymol retention (Figure 2). The samples added with the carrier system having a higher G:P ratio, and also higher total solid content (A2) showed a remarkable increase of thymol retention compared to the formulations with a small G:P ratio (A1). This fact is justified, since the diffusivities of the compounds through the wall, at higher solids content, should be lower [54,70]. On the other hand, the retention efficiencies for the NLCs were significantly higher than the spray-dried powders, showing values higher than 82%. One possible explanation for the high losses of EO in the dried product is probably associated with the small size of the lipid-based formulations droplets (nanoscale range), which increased significantly the surface area in contact with the spray-drying gas, favoring the evaporation of volatile compounds. Maybe the use of a more product friendly drying process, e.g., freeze-drying, would improve retention of volatiles in the dried powders. Besides the increase of the concentration of lipid materials would result in either the increase of the number of NLCs or their mean size, which could certainly contribute to the increase of the EO retention in the lipid matrices. However, the increase of NLCs concentration and/or mean size could increase the risk of particle aggregation during the spray-drying process.

**Figure 3:**
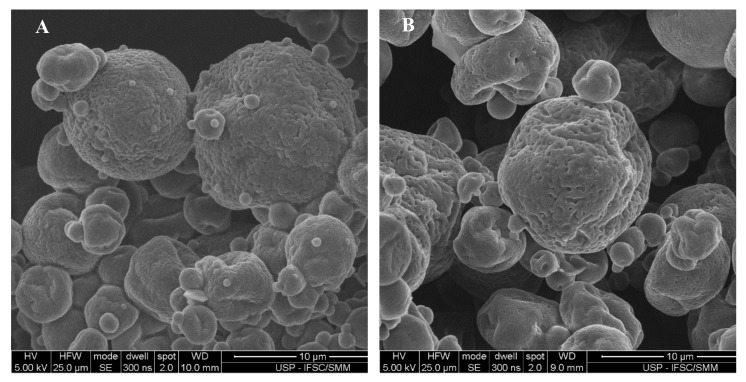
Typical morphology of spray-dried lipid-based formulations: F7A1 (**A**); F7A2 (**B**).

The morphology of the spray-dried particles is affected by factors as the drying kinetics and the constituents of the feed formulation [54]. Both the physical and chemical nature of the material being spray dried are important in determining its drying behavior and particle morphology [69]. The morphological appearance of the spray-dried particles (Figure 3) was found to have a round shape, heterogeneous size, and external surface with shriveled aspect, typical of spray-dried microcapsules [71] and very well consistent with previous studies [54,72]. With respect to the composition of the formulations, no significant difference could be observed in the particles’ morphology.

## 4. Conclusions

The results here reported show that the encapsulation of *Lippia sidoides* EO in NLCs is a feasible technology. These results are relevant indicating possible applications within the new area of the nutraceutical science [23,73,74,75]: nano nutraceuticals are a great challenge for the future, assuring their nutraceutical value at a nano-level as well as safety and efficacy [24,29,30]. The spray-drying of these lipid systems is promising, although demanding due to high product stickiness to the dryer (due the low melting point and also the glass transition temperatures), and losses of EO (low retention efficiency), exceeding 80% in some experimental runs. The samples added with the carrier system having a higher G:P ratio (and also higher total solid content—A2) showed a remarkable increase of thymol retention compared to the formulations with a small G:P ratio (A1). The differences between the physicochemical properties of the liquid formulations and redispersed dried products were not remarkable. The zeta potential and particle size of the liquid and redispersed dried product suffer effects of lipid matrix composition and drying carrier system, ranging from −16.4 mV to −31.6 mV and from 147 nm to 1531 nm; and from −25.9 mV to −30.9 mV, and from 525.3 nm to 1143 nm, respectively. The dried product present values of moisture content (from 3.78% to 5.20%) and water activity (<0.5) adequate to guarantee product stability. Furthermore, the powders presented low densities and exhibited I_Hausner_ (from 1.25 to 1.38), and I_Carr_ (from 25.0% to 37.93%), characteristic of weak and very weak flow. The dried product was promptly re-dispersible in water, returning its original consistency after adding water, which is an outstanding advantage, although optimization of EO load is still required.

## Figures and Tables

**Table 1:** Constituents of nanostructured lipid carriers (NLCs) (%, *w/w*).

Components	Function	Formulations							
		F5A1	F6A1	F7A1	F8A1	F5A2	F6A2	F7A2	F8A2
*Lippia sidoides* EO	Bioactive	3.0	3.0	3.0	3.0	3.0	3.0	3.0	3.0
Gelucire 50/13	Amphiphilic solid lipid	6.0	6.0	6.0	6.0	6.0	6.0	6.0	6.0
Oleic acid	Liquid lipid	1.0	1.0	-	-	1.0	1.0	-	-
Labrasol	Liquid lipid	-	-	1.0	1.0	-	-	1.0	1.0
Tween^®^ 80	Surfactant	0.35	-	0.35	-	0.35	-	0.35	-
Span^®^ 80	Surfactant	0.35	-	0.35	-	0.35	-	0.35	-
Poloxamer 188	Surfactant	-	0.7	-	0.7	-	0.7	-	0.7
Arabic gum	Carrier	3.0	3.0	3.0	3.0	8.6	8.6	8.6	8.6
Whey protein	Carrier	3.0	3.0	3.0	3.0	1.1	1.1	1.1	1.1
Aerosil 200	Carrier	-	-	-	-	1.1	1.1	1.1	1.1
Water	Solvent	83.3	83.3	83.3	83.3	121.5	121.5	121.5	121.5

**Table 2:** Constitution of *Lippia sidoides* essential oil (EO) identified by gas chromatography coupled to mass spectrometry (GC-MS).

Compounds ^a^	Relative Abundance (%) ^b^	KI (-) ^c^
α-thujene	0.95	928
Bicyclo[3.1.1]hept-2-ene, 2,6,6-trimethyl	0.56	937
2-β-pinene	0.16	980
β-myrcene	2.84	989
1-phellandrene	0.07	1007
*E*-β-ocimene	0.16	1011
α-terpinene	1.16	1018
Benzene, 1-methyl-4-(1-methylethyl)-	9.43	1025
Bornylene	0.64	1032
1.8-cineole	0.53	1033
1,3,6-octatriene, 3,7-dimethyl-, (*Z*)- (CAS)	0.11	1037
1,3,6-octatriene, 3,7-dimethyl-, (*E*)- (CAS)	0.16	1048
γ-terpinene	2.71	1060
Linalyl acetate	0.35	1099
2-(chloromethyl)tetrahydropyran	0.15	1145
Bicyclo[3.1.0]hex-3-en-2-one, 4-methyl-	0.26	1170
3-cyclohexen-1-ol, 4-methyl-1-(1-methylethyl)	0.68	1180
Thymol methyl ether	0.97	1231
Thymol	68.22	1297
α-copaene	0.34	1377
trans-caryophyllene	7.72	1420
Aromadendrene	0.46	1440
α- Caryophyllene	0.34	1455
Ledene	0.44	1493
δ-cadinene	0.10	1520
Caryophyllene oxide	0.43	1580

^a^ Compounds are listed in order of elution. ^b^ Relative abundance were calculated based on normalized MS peak areas, between the identified compounds. ^c^ KI: Kovats Index, a retention index relative to a series of alkanes (C10–C22).

**Table 3:** Properties of the spray-dried powders.

Form	Moisture Content (%)	Water Activity (-)	ρ_b_ (g cm^−3^)	ρ_t,1250_ (g cm^−3^)	I_Hausner_ (-)	I_Carr_ (-)
F5A1	3.78 ± 0.05	-	0.13	0.17	1.32	32.00
F6A1	4.05 ± 0.03	-	0.13	0.17	1.31	30.77
F7A1	4.21 ± 0.08	-	0.13	0.18	1.35	34.62
F8A1	4.42 ± 0.07	-	0.15	0.19	1.27	26.67
F5A2	4.45 ± 0.27	0.366 ± 0.011	0.17	0.22	1.33	33.33
F6A2	5.20 ± 0.08	0.421 ± 0.002	0.15	0.21	1.38	37.93
F7A2	4.40 ± 0.27	0.353 ± 0.011	0.17	0.23	1.34	33.93
F8A2	4.73 ± 0.02	0.353 ± 0.008	0.19	0.24	1.25	25.00
